# Identification of Risk Factors for Vascular Thrombosis May Reduce Early Renal Graft Loss: A Review of Recent Literature

**DOI:** 10.1155/2012/793461

**Published:** 2012-05-31

**Authors:** Anna Krarup Keller, Troels Munch Jorgensen, Bente Jespersen

**Affiliations:** ^1^Institute of Clinical Medicine, Aarhus University, Denmark; ^2^Department of Urology, Aarhus University Hospital, Brendstrupgaardsvej 100, Skejby, 8200 Aarhus N, Denmark; ^3^Department of Nephrology, Aarhus University Hospital, Brendstrupgaardsvej 100, Skejby, 8200 Aarhus N, Denmark

## Abstract

Renal graft survival has improved over the past years, mainly owing to better immunosuppression. Vascular thrombosis, though rare, therefore accounts for up to one third of early graft loss. We assess current literature on transplantation, identify thrombosis risk factors, and discuss means of avoiding thrombotic events and saving thrombosed grafts. The incidence of arterial thrombosis was reported to 0.2–7.5% and venous thrombosis 0.1–8.2%, with the highest incidence among children and infants, and the lowest in living donor reports. The most significant risk factors for developing thrombosis were donor-age below 6 or above 60 years, or recipient-age below 5-6 years, per- or postoperative hemodynamic instability, peritoneal dialysis, diabetic nephropathy, a history of thrombosis, deceased donor, or >24 hours cold ischemia. Multiple arteries were not a risk factor, and a right kidney graft was most often reported not to be. Given the thrombosed kidney graft is diagnosed in time, salvage is possible by urgent reoperation and thrombectomy. Despite meticulous attentions to reduce thrombotic risk factors, thrombosis cannot be entirely prevented and means to an early detection of this complication is desirable in order to save the kidneys through prompt reoperation. Microdialysis may be a new tool for this.

## 1. Background

In 1905, the first paper on kidney transplantation was published [1]. The French surgeon Alexis Carrel had, in the preceding years, developed techniques for vascular suture and anastomoses. In 1902, he transplanted a kidney from a small-size dog into its neck with the renal artery joined to the carotid artery and the vein to the external jugular vein. After three days, he noted a slightly greater blood flow than in the normal kidney and a urine production that was five times greater in the transplanted kidney. The animal died from an infection within few days. In 1912, Alexis Carrel received the Nobel Prize for his work.

Fifty years after the pioneering experiments, the first successful human renal transplantation was carried out in Boston by Dr. Joseph Murray in 1954 [2]. The donor was the patient's identical twin brother. Preceding the operation, monozygosity was confirmed by the successful exchange of a full thickness skin graft between the brothers. The transplanted kidney functioned for eight years until cardiac death of the patient. In the succeeding decades, a tremendous development has been taking place, with an increasing understanding of immunological aspects of transplantation and the opportunity for pharmacological immunosuppression, which was revolutionized after introduction of cyclosporine in the late seventies. In 1990, Joseph E. Murray and E. Donnall Thomas received the Nobel Prize for their discoveries concerning organ and cell transplantation in the treatment of human disease.

In 2012, kidney transplantation remains the best treatment of end-stage renal disease (ESRD) resulting in improved health and quality of life [3] as well as decreased health expenses compared to sustained dialysis [4]. Renal graft survival has improved over the past years, mainly owing to better immunosuppression. As a consequence, although vascular thrombosis is a rare complication, it has become a major cause of early graft loss, accounting for up to one third of graft loss within one month [5] and up to 45–47% within two to three months [6]. In the pediatric NAPRTCS (North American Pediatric Renal Transplant Coorporative Study) cohort from 1996–2001, thrombosis was the most common cause of early graft loss [7].

## 2. Incidence of Vascular Thrombosis

The surgical technique has only seen minor changes since the first renal transplantations were accomplished. Usually the kidney is placed extraperitoneally in the iliac fossa with end-to-side anastomoses to the external iliac artery and vein. In small children and infants, however, this approach is often associated with an increased risk of thrombosis [8]. To avoid this, the kidney graft is anastomosed end-to-side to the distal abdominal aorta and caval vein.

Vascular thrombosis is a devastating complication because it most often leads to renal graft loss. Overall, the incidence of arterial thrombosis is reported to be 0.2–7.5% and venous thrombosis to be 0.1–8.2% as seen from Tables 1 and 2 with the highest incidence among children and infants and lowest in reports with only living donors. The incidence range is wide and many studies are not comparable due to differences in composition of recipients and donors, such as different percentage of deceased donors.

In one of the studies with the lowest number of thrombosis [17], only living donors were used and furthermore the recipients were selected with an exclusion of diabetic patients. The pediatric patients from this cohort were later examined [35, 37] with no thromboses reported. This is unusual but could partly be due to selection of low risk donors between 21–60 years of age. In addition, there were no transplanted children below 5 years of age. In contrast, a subanalysis of NAPRTCS [26] revealed thrombosis-caused graft loss in 1.7% of all children receiving kidneys from a living donor. In the children less than 6 years of age, the incidence was 6.7%. These numbers are probably more reliable because NAPRTCS includes children of all ages and prospectively collected data on all pediatric patients in 73 centers [7].

Unfortunately, only few of the study data available are prospectively collected [7, 11, 19, 26, 28, 29, 31, 34], while most of them are retrospective. Five studies were named “consecutive case series” without thorough description of data collection [6, 9, 15, 17, 35]. This increases risk of bias and missing data. However, as thrombosis is an early complication that ultimately leads to graft loss in most cases, it is unlikely that it will go unnoticed. The question is of course, whether the cause of graft failure is correctly recorded in the medical chart. Three case-control studies are included in Table 1. Two of these included all cases from prospectively collected multicenter studies; Penny et al. [5] found 134 index cases of thrombosis in the Australian and New Zealand database, they selected an institutional control—which was the transplantation preceding the index case in the same institution and a donor graft control, the recipient of the kidney contralateral of the index case. Ojo et al. [13] identified 751 cases of thrombosis within the first 30 days in the prospectively collected United Network for Organ Sharing (UNOS) database, only 8 (1.1%) had to be excluded due to missing data. The cases were individually matched with two controls from the same year and the same transplantation center. The third case-control study is from a single center with the contralateral kidney as the only control [25]; the thrombosis case was solely included when the other kidney was transplanted in the same center thus leading to many exclusions and only 24 of 42 (57%) cases available for analysis.

## 3. Donors Risk Factors for the Development of Vascular Thrombosis

Historically, renal grafts with multiple arteries have been associated with an increased risk of thrombosis [38]; however, this does not seem to be the case in the modern transplant era [6, 12, 15, 17, 18, 21, 22, 27, 30, 32] where even a kidney with six arteries was successfully implanted [39]. Only one retrospective study from Iran reports significantly more surgical complications after multiple vessel kidney transplantation compared with recipients of single-artery graft [24]. In their statistical analyzes, they have analyzed hemorrhage, artery stenosis, renal artery thrombosis, and renal vein thrombosis all together, with the latter two being least frequent. It has previously been reported that multiple arteries is an independent risk factor for hemorrhage [17], ultimately leading to graft nephrectomy in 22% of the cases. This factor may carry weight in the first mentioned analysis, as they had a higher incidence of hemorrhage: 6.1% [24] compared to 1.9% in the latter [17]. No separate analysis for thromboses alone was provided from the Iranian center.

Surplus renal arteries are found in 10–20% of the population. In order to succeed in overcoming the technical challenges, it requires an experienced surgeon with an in-depth knowledge of possible variations in renal arterial anatomy and of microsurgical techniques for vascular reconstruction [39]. The arteries can either be anastomosed individually, in a Carrel patch (deceased donors), or they can be converted to a single artery, which will increase bench surgery time but decrease operative time for anastomosis.

There is no general consensus about whether a right renal graft has increased thrombosis risk compared to the left. A few centers, with both high and low thrombosis incidence, find no association [15, 17, 32], while others report a dramatic difference [6, 14, 25]. A recent publication from Spain [25] identified all kidney grafts thrombosed within 30 days after transplantation. The transplantation outcome of the contralateral kidney from the same donor was also identified and served as control. Twenty-four kidney pairs were analyzed and revealed no significant differences in recipients' characteristics, but a huge difference in graft sides, with 21 thrombosed right kidney graft (87.5%) versus three left kidneys. This corresponds to the results from a center in The Netherlands [6], where 85% of the thrombosed kidneys were right versus 58% of the controls without thrombosis. The right kidney has a short vein and long artery, which can make it more difficult to position. The artery can easily be kinked and the vein is prone to compression by the kidney. This is supported by a significant association between right kidney and technical surgical problems in a multivariate analysis [6]. Like problems with multiple vessels can be overcome; right kidneys may not compose such a substantial risk factor, when operated by a meticulous surgeon.

 The renal graft is often positioned in the contralateral iliac fossa, but also the same side as the original position can be used as there is no association between complications and the side of graft placement [6, 12, 15, 17].

On average, around 35% of all renal transplantations are carried out with a kidney from a living donor with large variation from country to country. When using a living donor, the anatomy is well known preoperatively and the procedure is scheduled. This often leads to better results and fewer thromboses [7], which is also evident from Tables 1 and 2. Many studies do not demonstrate an increased risk of thrombosis after receiving a kidney from a deceased donor compared to a living donor [12, 13, 15, 24, 30, 32]. This may be due to relatively small study populations with a different mix of donor compositions. However, in the NAPRTCS reports the overall thrombosis incidence has not declined in spite of a higher fraction of living donors in recent years [7, 26]. On the other hand, the question has been raised weather laparoscopic nephrectomy in fact comprises a risk factor. A large register study demonstrated significantly more frequent delayed graft function and acute rejection in children after laparoscopic compared to open living donor nephrectomy [40, 41]. However, this has been questioned in a recent publication from three large centers, where similar results for open and laparoscopic procedure have been found [42]. In both series, there have been a minimal number of thromboses and hence no statistical conclusions can be made.

A female donor doubles the risk of thrombosis in the case-control study from the Australian and New Zealand Dialysis and Transplant Registry [5], probably due to smaller vessel size, as suggested by the authors. However, this has not been confirmed in other studies [6, 13, 15, 27, 32].

Although few studies find no association between donor age and thrombosis [6, 12, 17], convincing studies point to the contrary [5, 7, 14, 15, 32]. There is no consensus on how the age analyzes should be performed, which may explain some of the dispute. In the former retrospective studies that found no association, one is analyzed for donor age >45 or <45 years [12], another used no donors from extreme age groups [17], and the last one did not divide into smaller age groups before analyzing and found no association within an adult donor population [6]. When stratified into age groups, a poorer outcome is found when the donors are very young [7, 14, 32], especially when less than six years of age [7, 14] or the recipient is a child. If the renal grafts are <7 cm in length, they are often transplanted en bloc into the recipient [43]. However, kidneys grow fast when transplanted into a larger recipient [43], and it may thus be desirable to transplant the kidneys individually whenever possible. Also donors >60 years increase the thrombosis risk [14], which might be connected with more atherosclerotic vessels (Table 3).

Warm ischemic time (WIT) has not been found to be a risk factor for thrombosis development [6, 13, 15, 17, 21]. This should be interpreted with caution, as it is very likely to be caused by similar low WIT for most kidneys. Cold ischemic time (CIT) varies more, and this is also reflected in the risk analyzes. Many studies state no increased risk with increasing CIT [6, 12, 13, 15, 21, 25, 30], often these reports have relatively low CIT at a mean of 20-21 hours [21, 25]. Other reports with fairly larger study populations state a higher thrombosis incidence with longer CIT [5, 7, 14, 32], especially when it exceeds 24 hours [7, 32]. This is based on univariate analyzes and little information is provided on the reasons for extended CIT, hence confounding events cannot be ruled out.

## 4. Recipient Risk Factors for Development of Vascular Thrombosis

As previously mentioned, the placement of the renal graft in small children may influence the thrombotic risk. In a retrospective German study, children aged 6 years or younger had kidney grafts transplanted either retroperitoneally to the iliac vessels in the fossa iliaca or extraperitoneally with end-to-side anastomoses to the distal aorta and caval vein [8]. The procedure was switched from the former to the latter in 1990, and when there was an equal number in both groups the outcomes were analyzed. Anastomoses to the iliac vessels strongly increased the risk of thrombosis, which occurred in 25.8% of the patients versus 3.3% of the children operated with aortic and caval anastomoses. In contrast, another study found no association between placement of the graft and loss due to thrombosis, but they included children up to 16 years of age and gave no separate analysis for the youngest children [32]. Although the aortic/caval approach is now accepted as the best for the small children, it is still debated whether trans- or extraperitoneal access is to be favored. Seemingly, extraperitoneal placement provides similar graft outcome, and it limits gastrointestinal complications considerably [44], hence this approach is used in most centers.

Intra- and postoperative hemodynamic instability is rarely mentioned in transplantation outcome reports, maybe because the data are not always accessible. Nevertheless, this may be an important contributor to thrombotic risk. In a retrospective report from The Netherlands, it was found to be an independent risk factor [6]. It was defined as period(s) of hypotension, mean arterial blood pressure ≤70 mmHg, for more than 10 minutes intraoperatively or during the first 48 hours postoperatively. This was the case among 32.5% of the patients who later suffered from acute vascular thrombosis, while only 4.5% of the controls had hypotensive period(s). 33.3% (11/33) of hemodynamic instable patients developed thrombosis and lost their graft. This has been described previously in another study, where one of twelve arterial thromboses was explained by intraoperative hypotension with visibly poor perfusion to the kidney [9]. A pediatric study suggests hemodynamic instability to be even more important in children and find the young child more prone to nontechnical thrombosis because of a relatively low cardiac output, particularly in relation to a much larger kidney that leads to less efficient renal perfusion [45], hence an aggressive fluid therapy and cardiovascular monitoring are crucial.

Due to transplantation being a relatively infrequent operation in many centers, some reports only have few patients of extreme ages and a difference was not always found [6, 12, 13, 17, 25]. In a NAPRTCS report with a substantial number of very young recipients down to 0-1 years of age together with older children, age was only approaching significance as a predictor of graft thrombosis [7]. In other pediatric reports, the patients developing a thrombosed graft are younger [27], usually below 5–6 years [26, 32]. Yet, excellent pediatric outcome with no thromboses is seen in some centers [46, 47], even though a high proportion of the children have been very young. In both reports, some of the success is attributed to a very aggressive fluid management, and they underscore the critical importance of appropriate fluid resuscitative measures in small recipients of adult-sized kidneys [47]. A minimum recipient weight of 5, preferable 10 kg is suggested prior to operation [36]. The operative technique is based on the weight and not the age of the child.

Also elderly recipients are more prone to graft thrombosis [48]. Nevertheless, it is becoming more common to transplant patients at older ages and hence, in 2007 up to 18.9% of transplantations in American centers were performed in patients >65 years. When the patients were appropriately selected, a short time graft survival similar to that of other adults was noted [49]. This was also the conclusion in another study, where they propose that chronological age barriers should be substituted by biological limits [50].

In the preceding paragraph, it was concluded that donor gender was of less importance. The same is the case for recipient gender, with only one large case-control study finding a 46% higher likelihood of thrombosis in women [13], while several others report no association [6, 7, 15, 17, 24, 25, 32]. Overall, 54–74% of reported recipients are male, in agreement with a higher prevalence of end-stage renal disease (ESRD) in men [51].

The underlying disease that causes ESRD in the patient also plays a role in the transplantation outcome. Of significance is diabetic nephropathy [6, 13, 15] with an increased thrombotic risk. This might be because of diabetic angiopathy, as also atherosclerosis of either the recipient or donor vessels increases the risk [21, 22]. However, a Spanish case-control study with rather few subjects in the analysis found no association with diabetes mellitus [25] and another found no connection to any underlying disease [5]. Systemic lupus erythematosus increases the thrombosis risk compared to hypertensive nephropathy [13]. This is most probable due to the antiphospholipid antibody syndrome, which has been pointed out earlier [52].

The progression of the renal disease and possible comorbidities determine when and how the patient should be treated. There are three renal replacement options when reaching ESRD: transplantation, hemodialysis (HD), and peritoneal dialysis (PD). The choice, order, and duration of treatment can have a substantial impact on transplantation outcome. Preemptive transplantation, that is, when the new kidney is transplanted before initiation of dialysis, while the native kidneys still have a high urine production, is preferred to avoid dialysis and associated especially cardiovascular risks, but it has been found to be associated with increased thrombosis risk compared to transplantation after initiation of HD [7, 13]. Furthermore, a high native urine production in combination with dialysis is a risk factor [27]. It is speculated that these patients may be more difficult to keep in a relatively volume-expanded state in the intra- and postoperatively. Preemptive transplantation is favored whenever possible, as its superior long-term graft survival is well established [53, 54]. However, more focus is warranted on aggressive and well-monitored fluid management of patients undergoing transplantation and preoperative stop of medical treatment like diuretics and antihypertensive drugs except for beta blockers.

Several studies with large patient cohorts find substantial more thrombotic graft loss among patients who have been on PD prior to transplantation compared to HD [7, 10, 13, 55], while just a few retrospective studies state the type of dialysis not to affect the graft outcome [6, 14]. An acquired thrombophilic state may be involved, possibly due to selective protein loss in the peritoneal fluid, like in nephrotic syndrome. Another contributing factor could be selection of patients; they might have been switched from HD to PD because of vascular access problems, which may indicate atherosclerosis. At least this switch has been found to increase risk more than PD in itself [13]. In a retrospective study, thrombosis has been reported to comprise up to 41% of early graft failure among PD patients and only 30% among HD patients [55]. Antithrombotic treatment of HD patients during sessions or a more efficient dialysis may contribute as well. However, these observations should be carefully interpreted, as the data for the cause of early graft loss were available from only one third of the cases in this study.

Inherited or acquired forms of thrombophilia increase the risk of thrombosis in general and hence also in a newly received renal graft and anticoagulation may be a sound consideration. Screening for thrombophilia has shown to decrease the number of thrombotic events [34, 56]. This can be done in different ways: one study has screened blood samples of all patients prior to transplantation [34], while another screened the patients by registration of history of prior thrombosis including deep venous thrombosis, multiple miscarriages, or family history of thrombosis among others [56]. Subsequently, these patients had thrombophilia confirmed by laboratory tests. Two patients not initially identified by this method developed thrombotic complications and had a hypercoagulable disorder documented afterward. Transplantation of recipients with nephrotic syndrome is a risk factor for thrombosis, particularly in small recipients where nephrectomy prior to transplantation should be considered.

Some studies are too underpowered to detect an effect of anticoagulation among unselected recipients. For instance, heparin is used prophylactic in case of vague risk factors [17], but it has also been shown not to affect the outcome [32]. In the latter study, only 254 patients were included, even though their power calculation required at least 1,500 patients in each arm. Furthermore, the patients were not randomized to heparin or not, but by 1994 heparin was implemented as routine treatment, and the patients were divided for analysis according to transplant year. Unfortunately, this kind of power calculation is rarely stated in transplantation reports, and few studies are prospective with randomization. Aspirin has been suggested to be a more convincing preventer of thrombosis [11, 57]. Nevertheless, this should be carefully administered as it increases the bleeding risk when graft biopsy is needed at short notice. To overcome this, low molecular weight heparin can be initiated perioperatively and used during the time when renal graft biopsy should be an option. Also administration of rabbit antithymocyte globulin in a pediatric population decreased the platelet count and reduced thrombosis risk significantly [31]. The distribution of thrombotic risk factors was similar in the groups, but it was not a randomized study.

Delayed graft function is strongly correlated to thrombosis and a vascular cause for decreased graft function should always be considered [6, 14, 21]. Neither the degree of HLA mismatch [5, 13, 25] nor immunosuppression with cyclosporine [5, 6, 26, 27] was correlated to thrombotic events.

## 5. Graft Salvage Is Possible

Diagnosis of vascular thrombosis can be time consuming, from request to implementation, and may thus be delayed. Consequently, kidney grafts developing thrombosis are most often lost, and the patient is returned to dialysis treatment. However, with early diagnosis and intervention, it is possible to save the kidney [16, 20, 33, 58–60]. Even up to 15–20 hours after onset of symptoms, salvage has been reported [61, 62]. Flow in the occluded vessel can be reestablished by open surgery with urgent thrombectomy [16, 33]. If the thrombosis is a late complication, local thrombolysis by an endovascular access [58, 59] may be tried; this is, however, most likely more risky in a recently operated patient with an increased risk of bleeding.

## 6. Methods for Diagnosis of Vascular Complications

Venous thrombosis classically presents itself with a swollen tender graft and hematuria, while arterial thrombosis can be more silent with a sudden drop in urine output. Intrarenal thrombosis may also be involved in severe rejections. Symptoms are often sparse, and the risk of delayed graft function is high. This can be masked by a prior low urine production or native kidney function in preemptive patients. Usually thrombotic events occur within the first 10 postoperative days, in 93% of the cases within day seven [5]. Arterial thrombosis is often presented later than venous thrombosis [14], but the latter can also occur as a late complication.

When vascular thrombosis is suspected clinically or in a routine screening, the diagnosis is confirmed by color Doppler flow examinations, which may also be used within the first hours after transplantation if the kidney is not functioning at once after revascularization. This technique detects vascular complications in general, including renal artery stenosis, with an overall sensitivity and specificity of 88% and 85%, respectively [63]. The accuracy of complete vascular thrombosis detection is higher but, nevertheless, false-positive as well as false-negative results can still be seen [64], and the diagnosis is often made too late to salvage the transplanted kidney, also due to delay from request to realization of the procedure. It is a static method that most often has to be repeated in patients with delayed graft function [65], because it is impossible to perform both ultrasound and biopsies at intervals frequent enough for safe early warnings, and these patients are at a higher risk of thrombosis, as mentioned above. When the thrombosed kidney is removed, it should be histopathologically examined to exclude rejection as the primary event [21]. During salvage surgery, a renal biopsy is often requested.

## 7. Reducing Early Renal Graft Loss

Despite meticulous attentions to the risk factors discussed, thrombosis is not completely preventable and means to a fast early detection of this complication are desirable in order to implement the above-mentioned opportunities for salvage. Some steps have been taken in this direction, and thermodiffusion has been suggested as a possible new dynamic diagnostic technique. A small thermodiffusion electrode placed in the tissue continuously measures the absolute microcirculation in mL/100 g tissue per minute. It has been applied to ischemic porcine kidneys [66] and livers [67]. Furthermore, it has been clinically applied to thirty renal transplant grafts [68]. All studies found a significantly lower microcirculation in the organs subjected to most ischemic damage, but this technique still needs further validation, and there have been no new publications on the subject since 2004.

Microdialysis is another minimally invasive technique for dynamic monitoring of the local metabolism, which is being increasingly used experimentally as well as in the clinic. A small catheter is placed in the tissue of interest and by simple diffusion through a semipermeable membrane; the concentration of local metabolites can be estimated. The reconstructive surgeons have used it for more than ten years for free flap monitoring, for example, when the esophagus is reconstructed by a free jejunal flap after carcinoma resection. Critical ischemia locally within the flap is detected by microdialysis; accordingly, the flap can be reoperated and revascularized in time [69]. The two-lumen catheter is continuously perfused and samples are taken every 30 or 60 minutes for analysis with results provided within minutes. The free flap is monitored for 4–7 days after surgery; this timeframe is also relevant following renal transplantation, where 93% of thromboses occur within the first 7 days [5]. Up till February 2012, more than 14,000 studies on microdialysis have been published; of these 183 have been made on kidneys including recent publications on renal ischemia. In pigs, microdialysis detects renal ischemia within 10–30 minutes, while metabolites remain at stable levels when no ischemia is present. This has been closely studied in different animal models of total ischemia [70–72], partial ischemia [73], autotransplantation with subsequent postoperative ischemia [74], or transplantation with risk of thrombosis [75]. All studies had equal conclusions, with a fast detection of renal ischemia by use of microdialysis.The first investigations on human kidneys undergoing nephrectomy due to cancer [76] have recently been carried out. They found similar results, indicating that a clinical application of the technique seems feasible.

## 8. Conclusions

Arterial and venous renal graft thromboses are rare, but nevertheless devastating complications that usually lead to graft loss and accounts for a considerable fraction of early graft losses. Especially in the situation with delayed graft function, there is a need for continuous monitoring to facilitate salvage and avoid explorative surgery when no thrombosis is present.

The most significant risk factors for thrombosis are donor-age below 6 or above 60 years or recipient-age below 5–6 years, per- or postoperatively hemodynamic instability, PD, diabetic nephropathy, a history of thrombosis, deceased donor, or more than 24 hours CIT. Multiple arteries and right kidney are not as severe risk factors as earlier thought, but should, nevertheless, be handled very carefully by the surgeon. A lot of the risk factors are not adjustable, and the kidneys are to be used as there is universal organ shortage and a need for kidneys even from marginal donors. When risk factors are present, the number of thrombotic events may be reduced, for example, by aggressive fluid management of pediatric patients or anticoagulation of thrombophilic patients. Furthermore, there is a need for early detection as thrombosis is not completely preventable. Microdialysis may be suitable for this.

## Figures and Tables

**Table 1:** RAT: Renal artery thrombosis, RVT: renal vein thrombosis, ^*¤*^: mean, ∗: % deceased, ∗∗: see text, c.s.: case series, c.r.: chart review, N/A: not available.

Author	Nationality	Year	*N*	Recipient age	Donor*	RAT + RVT	RAT	RVT	Study design
Jordan [9]	Canada, Toronto	1970–1980	341	13–67	84	4.4% (15)	3.5% (12)	0.9% (3)	Consecutive c.s.
Penny [5]	Australia, ANZDATA	1980–1992	6153	N/A	N/A	2.2% (134)	1.1% (70)	1.0% (64)	Case-control**
Murphy [10]	Ireland, Belfast	1989–1992	202	adults	N/A	4.5% (9)	1.0% (2)	3.5% (7)	Retrospective
Stechman [11]	UK, Oxford	1997–1992	401	27–75	78	1.0% (4)	0.75% (3)	0.25% (1)	Prospective
Benedetti [12]	USA, Minnesota	1985–1993	998	16–74	51	1.4% (14)	0.4% (4)	1.0% (10)	Retrospective
Bakir [6]	Holland, Groningen	1986–1994	558	14–71	100	6.1% (34)	2.0% (11)	3.4% (19)	Consecutive c.s.
Ojo [13]	USA, UNOS	1990–1996	84513	>18	N/A	0.9% (751)	N/A	N/A	Case-control**
Perez [14]	Spain, A Coruña	1988–1997	827	44**^*¤*^**	100	5.7% (47)	2.3% (19)	3.4% (28)	Retrospective
Englesbe [15]	USA, Michigan	1993–1997	714	all	43	1.82% (13)	0.8% (6)	1.0% (7)	Consecutive c.s.
Samhan [16]	Kuwait, Hawaly	1993–1998	151	all	24	3.3% (5)	2.6% (4)	0.7% (1)	N/A
Osman [17]	Egypt, Mansoura	1976–1999	1200	5–62	0	0.5% (12)	0.4% (5)	0.1% (1)	Consecutive c.s.
Mazzucchi [18]	Brazil, Sao Paulo	1995–1999	356	18–70	64	1.4% (5)	0.3% (1)	1.12% (4)	Retrospective c.r.
Parada [19]	Portugal, Coimbra	1980–2001	1000	41**^*¤*^**	98	0.9% (9)	0.6% (6)	0.3% (3)	Prospective
Orlic [20]	Croatia, Rijka	1971–2002	725	N/A	53	1.1% (8)	0.6% (4)	0.6% (4)	N/A
Hernández [21]	Spain, Tenerife	1996–2004	870	18–76	100	4.8% (42)	3% (26)	1.8% (16)	Retrospective c.r.
Sanni [22]	UK, Newcastle	1990–2005	1308	N/A	93	2.8% (36)	N/A	N/A	Retrospective
Dimitroulis [23]	Greece, Athens	1980–2005	1367	N/A	44	2.3% (31)	2% (27)	0.3% (7)	Consecutive
Salehipour [24]	Iran, Shiraz	1988–2006	1500	4–70	20	1.1% (16)	0.6% (9)	0.5% (7)	Retrospective c.r.
Amézquita [25]	Spain, Madrid	1990–2006	772	N/A	100	5.5% (42)	N/A	N/A	Case-control**

**Table 2:** Pediatric patients: RAT: Renal artery thrombosis, RVT: Renal vein thrombosis, ∗: % deceased, mc: multicenter, c.s.: case series, N/A: not available.

Author	Nationality	Year	*N*	Recipient age	Donor*	RAT + RVT	RAT	RVT	Study design
Harmon [26]	USA, NAPRTCS	1987–1989	1045	0–17	54	2.6% (27)	N/A	N/A	Prospective, mc.
van Lieburg [27]	Holland, Nijmegen	1977–1990	100	children	93	12% (12)	4% (4)	7% (7)	Retrospective
McEnery [28]	USA; NAPRTCS	1987–1992	2193	0–17	55	3.2% (71)	N/A	N/A	Prospective, mc.
Johnson [29]	UK, Ireland	1986–1995	1252	<18	100	4.0% (50)	N/A	N/A	Prospective, mc.
Ismail [30]	Poland, Warsaw	1984–1995	176	1–18	92	4.0% (7)	1.7% (3)	2.7% (4)	Retrospective
Kamel [31]	Ireland, Dublin	1986–1998	120	1–17	100	5% (6)	0.8% (1)	4.2% (5)	Prospective
Adams [8]	Germany, Heidelberg	1977–1998	61	1–6	77	13.1% (8)	4.9% (3)	8.2% (5)	Retrospective
Nagra [32]	UK, London	1987–2000	254	1–16	75	9.8% (25)	N/A	N/A	Retrospective
McDonald [7]	USA; NAPRTCS	1987–2001	7247	0–17	47	2.7% (199)	N/A	N/A	Prospective, mc.
Mickelson [33]	Canada, Vancouver	1984–2003	24	1–6	71	4.2% (1)	4.2% (1)	N/A	Retrospective
Kranz [34]	Germany, Essen	1998–2003	66	children	74	0	0	0	Prospective
El-Husseini [35]	Egypt, Mansoura	1976–2004	216	5–18	0	0	0	0	Consecutive c.s.
Garcia [36]	Brazil, Porto Alegre	1989–2005	40	1–5	25	10% (4)	7.5% (3)	2.5% (1)	N/A

**Table 3:** Factors associated with increased risk of thrombosis.

Increased risk of thrombosis	
Donors	Recipients
Donor age >60	Recipient age <5–6 or >50
Young donors <6 years	Peritoneal dialysis
Cold ischemic time >24 h	Recipient diabetes mellitus
Renal vessel atherosclerosis	Renal vessel atherosclerosis
Right kidney	History of thrombosis
	Technical surgical problems
	Illiac graft anastomosis, age <6 years
	Hemodynamic instability
	No use of aspirin
	Delayed graft function

## Abbreviations

NAPRTCS: North American Pediatric Renal Transplant Corporative Study
UNOS: United Network for Organ Sharing
WIT: Warm ischemic time
CIT: Cold ischemia time
HD: Hemodialysis
PD: Peritoneal dialysis
TX: Transplantation
ESRD: End-stage renal disease.
